# The impact of Public Reporting on clinical outcomes: a systematic review and meta-analysis

**DOI:** 10.1186/s12913-016-1543-y

**Published:** 2016-07-22

**Authors:** Paolo Campanella, Vladimir Vukovic, Paolo Parente, Adela Sulejmani, Walter Ricciardi, Maria Lucia Specchia

**Affiliations:** Department of Public Health, Section of Hygiene, Catholic University of Sacred Heart, L.go F. Vito 1, 00168 Rome, Italy

**Keywords:** Public reporting, Healthcare quality, Clinical outcomes, Systematic review

## Abstract

**Background:**

To assess both qualitatively and quantitatively the impact of Public Reporting (PR) on clinical outcomes, we carried out a systematic review of published studies on this topic.

**Methods:**

Pubmed, Web of Science and SCOPUS databases were searched to identify studies published from 1991 to 2014 that investigated the relationship between PR and clinical outcomes. Studies were considered eligible if they investigated the relationship between PR and clinical outcomes and comprehensively described the PR mechanism and the study design adopted. Among the clinical outcomes identified, meta-analysis was performed for overall mortality rate which quantitative data were exhaustively reported in a sufficient number of studies. Two reviewers conducted all data extraction independently and disagreements were resolved through discussion. The same reviewers evaluated also the quality of the studies using a GRADE approach.

**Results:**

Twenty-seven studies were included. Mainly, the effect of PR on clinical outcomes was positive. Meta-analysis regarding overall mortality included, in a context of high heterogeneity, 10 studies with a total of 1,840,401 experimental events and 3,670,446 control events and resulted in a RR of 0.85 (95 % CI, 0.79-0.92).

**Conclusions:**

The introduction of PR programs at different levels of the healthcare sector is a challenging but rewarding public health strategy. Existing research covering different clinical outcomes supports the idea that PR could, in fact, stimulate providers to improve healthcare quality.

**Electronic supplementary material:**

The online version of this article (doi:10.1186/s12913-016-1543-y) contains supplementary material, which is available to authorized users.

## Background

Public reporting (PR) is a mechanism of “providing data about a health care structure, process, or outcome publicly available or available to a broad audience free of charge or at a nominal cost, in order to be able to compare data across providers or to a national/regional data report on performance for which there are accepted standards or best practices” [1]. Public release of quality and clinical performance of the healthcare providers is becoming increasingly common among the healthcare systems worldwide. Policy and decision-makers in a demand-driven healthcare system are becoming more interested in having information about quality performance [2] and thereby PR has been proposed as a mechanism for providing more transparency and accountability of healthcare providers [3]. Constant improvement of the quality of care should be one of the top priorities of healthcare providers [4]. According to the theory of PR, healthcare users are expected to inform themselves about the quality of healthcare system before selecting the particular provider and so those with high performance would be rewarded by selecting, while low performers would be avoided and thereby stimulated to improve their performance [5–8].

There have been suggested several pathways through which quality might be improved after the release of performance data. First pathway, the selection pathway, proposed by Berwick et al., is based on the concern of healthcare providers about their market share, where consumers choosing better performers, motivate providers’ efforts to improve quality in order to attract more patients. The change pathway, also proposed by Berwick et al., is based on the concept that identifying quality deficits is sufficient to stimulate the professional motivation of clinicians and organizations to improve [9]. After observing in their study that these two pathways were a relatively weak stimulus to action, Hibbard et al., introduced the third pathway - the reputation pathway, based on the premise that providers which perform poorly, after being identified through the PR, suffer damage to their reputation and further motivate quality improvements in order to protect or improve reputation [10].

PR of clinical outcomes data is a great tool for increasing the transparency in healthcare which enables patients to make informed choices about their healthcare. The most convincing rationale for the PR of clinical outcomes is the one’s right to be aware of the quality of care that he/she is likely to receive from providers [11]. Cardiac surgery has been a pioneer field for the publication of clinical outcomes, since being among the most frequently performed complex surgical procedures [12]. The collection and publication of standardized military hospital mortality rates by Florence Nightingale in 1863 where highlighting the differences in mortality rates between hospitals is believed to be the earliest attempt of PR of clinical outcome and in general [13]. The modern practice of PR systems started in the late 1980s in the USA with the introduction of Coronary Artery Bypass Graft (CABG) report cards of New York State Department of Health, as the first state wide program where the risk adjusted post-operative mortality rates following CABG surgery are being published at the level of both the hospital and the individual surgeon resulting in a 41% decline in risk-adjusted CABG mortality rate [14]. In Europe, back in 1994, Scotland was the first to adopt PR with the Clinical Resource and Audit Group (CRAG) [15]. After these initiatives, other countries have started to follow their example and implement PR into their healthcare systems.

Several authors, over the years have studied the effects of PR on clinical outcomes and, still nowadays, there are inconsistent results in the literature. The review of healthcare PR by Fung et al. found mixed signals among the studies of the effect of PR on outcomes, with some studies showing no effects and others showing minimal effects. Indeed the authors concluded that a solid evidence is still lacking and the systematic evaluation of many major PR systems is needed [5]. The scarcity of a solid evidence does not necessarily suggests the lack of effect and, since the publication of Fung et al., many studies have been conducted to explore the effect of public release of performance data on clinical outcome, but always with incoherent results. Thus, we took the aim to perform an up-to-date systematic review of scientific literature in order to synthesize, both qualitatively and quantitatively, the impact of PR on clinical outcomes.

## Methods

A protocol was developed and a systematic review and a meta-analysis were conducted and reported in accord with PRISMA guidelines for meta-analyses and systematic reviews [16].

### Search strategy and study selection

A literature search was performed by accessing Pubmed, Thomson Reuters Web of Science and Scopus databases to identify studies that investigated the relationship between PR and clinical outcomes. The search terms “public reporting”, “quality reporting”, “information dissemination”, “data shar*” and “report card*” were used, by specifying “health” for databases that also covered non-health topics.

Our search was restricted to English language studies published from 1st January 1991 to 31st December 2014. Studies were considered eligible if they comprehensively described the PR mechanism in terms of subjects, setting, location and dissemination way, if the study design adopted was clearly described and if they investigated the relationship between PR and clinical outcomes. Studies not reporting original data as well as studies focusing only on non-clinical effects of PR were excluded.

Two reviewers independently screened titles and identified abstracts of relevant titles. Full texts of potential citations were subsequently obtained and independently screened by the two reviewers for inclusion. Disagreements were resolved through discussion. Additional relevant publications were identified from the references of the initially retrieved articles.

### Data extraction and analysis

From each study data on the first author’s last name, year of publication, objective, subject, setting, location, PR mechanism, clinical outcome assessed and key findings were extracted. For each clinical outcome assessed, quantitative data were also extracted if available.

Two reviewers conducted all data extraction independently and disagreements were resolved through discussion. The same reviewers evaluated also the quality of the studies using a GRADE derived approach (see Additional file 1) [17].

Among the clinical outcomes evaluated, meta-analysis was performed for overall mortality rate which quantitative data were exhaustively reported in 10 different studies. Because of the significant heterogeneity expected among the studies performed in different settings, the random effects model was employed using the Der Simonian and Laird’s method [18].

Heterogeneity was quantified using the Cochran Q test and I^2^ statistics [19] and subgroup analyses were performed for different study design and setting.

Sensitivity analyses were conducted by excluding one study at a time from the meta-analysis to determine whether the results of the meta-analysis were influenced by individual studies and whether risk estimates and heterogeneity were substantially modified.

The presence of publication bias was assessed using the Egger’s test [20].

All analyses were carried out using Review Manager, version 5.2.7 for Mac (The Nordic Cochrane Centre, Copenhagen, Denmark) and Stata, version 13.1 for Mac (StataCorp, College Station TX, USA).

## Results

### Characteristics of the studies

**Fig. 1:**
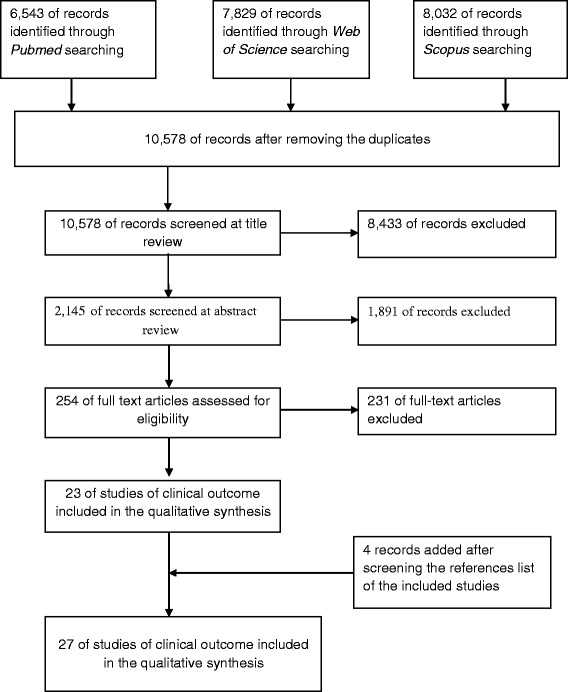
Flowchart depicting literature search and study selection

We identified a total number of 22,404 studies through Pubmed, Thomson Reuters Web of Science, Scopus online databases search. After removing the duplicates, 10,578 studies were left, and, carefully reading the titles, 2,145 studies were assessed for eligibility. Further step was reviewing the abstracts, and 254 full text articles were obtained. By not fulfilling the inclusion criteria, 231 full text articles were excluded, and 4 were individuated from the screening of references list of studies that fulfilled inclusion criteria, leaving 27 studies to be included in our analysis [14, 21–46]. Figure 1 depicts the process of literature search and study selection.

The publication years of the studies were ranged from 1994 [21] until the most recent ones, from 2014 [44–46]. Most of the 27 studies included in our review (*N* = 23) were carried out in the US [14, 21–30, 32–39, 41–44], one in Canada [31] and Italy [40], and the remaining two in China [45, 46]. Twelve were cohort studies in which the control and study cohorts of patients were taken from different facilities over the same period of time [23, 26, 29, 33, 35, 38, 39, 42–46], 14 were cohort studies in which the control and study cohorts of patients were taken from the same facilities before and after the introduction of a PR mechanism [14, 21, 22, 24, 25, 27, 28, 30–32, 34, 36, 37, 41], while the remaining study used both cohort designs [40]. The majority of the studies included in this review presented performance information in the form of “report cards”, while the others used different forms to communicate data to the public (Table 1).

**Table 1:** Characteristics of the included studies and effects of PR on clinical outcomes

First author, year	Objective	Study design	Subject, Setting and Location	Public Reporting mechanism	Clinical outcomes	Key findings	Effect on reported outcome
Dziuban et al., 1994 [21]	To evaluate the impact of NYS CSRS program in CABG related mortality in one hospital identified as poor performing	Cohort study for the same facility before and after the introduction of a PR mechanism	One poorly performing, high risk hospital in New York (1992–1993)	NYS CSRS	Cardiac mortality	The NYS CSRS program has been associated with a reduction in the actual CABG-related mortality from 3.52 % in 1989 to 2.78 % in 1992. The risk-adjusted mortality, using pooled data from 1989 to 1992, decreased from 4.17 % in 1989 to 2.45 % in 1992	Positive
Hannan et al., 1994 [14]	To examine changes in the risk-adjusted mortality and operation volume associated with CABG procedures performed during the first 4 years of NYS CSRS in three groups of hospitals	Cohort study for the same facility before and after the introduction of a PR mechanism	New York Hospital cardiac surgeons performing CABG (1989–1992)	NYS CSRS	Cardiac mortality and CABG operation volume	During 4 years of NYS CSRS program, the risk-adjusted mortality decreased from 2.72 to 2.19 % for group1, from 4.24 to 2.51 % for group 2 and from 7.12 to 2.77 % for group 3. The groups of providers that showed the highest initial mortalities manifested the most improvement. The volume of operations performed by the various provider groups did not change substantially in the 4-year period	Positive
Rosenthal et al., 1997 [22]	To measure changes in hospital mortality that occurred after implementation of the CHQC, which publicly released in-hospital mortality rates	Cohort study for the same facility before and after the introduction of a PR mechanism	Discharges with 8 diagnosis from Northeastern Ohio hospitals (1992–1993)	CHQC	Cardiac, Respiratory, Neurologic, Gastro-Intestinal mortality	Risk-adjusted mortality for most conditions declined for 3 subsequent periods after publication of mortality data (July-December 1992/January-June 1993/ July-December 1993). Decreases in mortality rates were statistically significant in weighted linear regression analyses for heart failure (0.50 % per period) and pneumonia (0.38 % per period).	Positive
Peterson et al., 1998 [23]	To examine the impact of the NYS CSRS on in-hospital mortality rates by comparing mortality rates in New York to those in other states	Cohort study among different facilities with and without PR mechanisms, over the same period of time	New York Hospital Medicare beneficiaries aged 65 years and older who underwent bypass surgery between (1987–1992)	NYS CSRS	Cardiac mortality	After NYS CSRS program initiation, unadjusted 30-day mortality rates following bypass declined by 33 % in NY Medicare patients compared with a 19 % decline nationwide. Risk-adjusted 30-day mortality of bypass surgery in NY patients declined an average of 10.30 % per year (1987–1992) compared with 5.80 % for patients in the rest of the nation.	Positive
Baker et al., 2002 [24]	To examine mortality trends during a period (1991–1997) when the CHQC program was operational in Cleveland Hospitals	Cohort study for the same facility before and after the introduction of a PR mechanism	Cleveland Hospital Medicare patients for cardiac, respiratory, neurologic or gastro-intestinal diseases (1991–1997)	CHQC	Cardiac, Respiratory, Neurologic and Gastro-intestinal mortality (GIH)	During CHQC program risk-adjusted in-hospital mortality declined for all conditions except stroke and GIH. The 30-day mortality declined significantly only for CHF to 1.40 %, and COPD to 1.60 %. For stroke, risk-adjusted 30-day mortality actually increased by 4.30 %.	Mixed
Chassin, 2002 [25]	To examine the impact of NYS CSRS implementation on mortality rate outlier status and CABG mortality	Cohort study for the same facility before and after the introduction of a PR mechanism	Lowest And Highest CABG Mortality Hospitals In New York, (1989–1995)	NYS CSRS	Cardiac mortality	After NYS CSRS program was implemented risk-adjusted mortality fell 41 % statewide in New York. Mortality statewide continued to fall in the next period; the crude mortality reached 2.15 % in 1998 from 3.52 % in 1989.	Positive
Clough et al., 2002 [26]	To verify the decline in in-patient mortality in Cleveland Hospitals and to better understand relationship with CHQC project	Cohort study among different facilities with and without PR mechanisms, over the same period of time	Hospitals included in the Ohio Hospital Association’s in-patient discharge data (1992–1995)	CHQC	Cardiac, Respiratory, Neurologic and Gastro-intestinal mortality	No significant beneficial effect of the CHQC project on hospital mortality in Cleveland was demonstrated. The rate of decline in mortality in Cleveland (−0.218 % per six months) was statistically indistinguishable from that in the rest of the state.	None
Baker et al., 2003 [27]	To describe trends in risk-adjusted mortality for six acute conditions for hospitals that were identified as outliers by CHQC compared with other hospitals.	Cohort study for the same facility before and after the introduction of a PR mechanism	Medicare patients with AMI, heart failure, gastrointestinal hemorrhage, obstructive pulmonary disease, pneumonia, or stroke receiving care at Cleveland-area hospitals (1991–1997)	CHQC	Cardiac, Respiratory, Neurologic and Gastro-intestinal mortality	Hospital outlier status was not significantly related to changes in risk-adjusted 30-day mortality. During CHQC reporting period, the absolute decline in risk-adjusted 30-day mortality at “average” hospitals was 0.50 %.	None
Dranove et al., 2003 [28]	To study the effects of PR in New York and Pennsylvania on health care providers and patient outcomes	Cohort study for the same facility before and after the introduction of a PR mechanism	Medicare beneficiaries and hospitals found in a Medicare claims data set and hospitals participating in the American Hospital Association annual survey (1987–1994)	New York and Pennsylvania CABG report card	Cardiac, Respiratory, Neurologic and Gastro-intestinal mortality and readmission	Report card provided statistically marginal evidence that the average mortality rate in NY and PA increased by 0,45 % point on a base of 33 %. Report cards increased significantly the average rate of readmission with heart failure by approximately 0,50 % point.	Negative
Moscucci et al., 2005 [29]	To compare in-hospital mortality from large multicenter PCI databases in Michigan, where PR is not mandated, and in New York where PR of PCI data is mandatory	Cohort study among different facilities with and without PR mechanisms, over the same period of time	Patients included in a multicenter PCI database in Michigan Hospitals and statewide PCI database in New York Hospitals (1998–2000)	PCI database in Michigan and New York	Cardiac mortality	The unadjusted in-hospital mortality rate was significantly lower in New York than in Michigan (0.83 % vs. 1.54 %, OR = 0.54). However, after adjustment for comorbidities, there was no significant difference in mortality between the two groups (adjusted OR = 1.05).	None
Carey et al., 2006 [30]	To examine the relationship between CCSIP and cardiac surgery mortality in California Hospital	Cohort study for the same facility before and after the introduction of a PR mechanism	Cardiac surgery patients (CABG, PCI, Valve) in California Hospital (1998–2004)	CCSIP	Cardiac mortality	The risk-adjusted in-hospital mortality for CABG decreased and PCI mortality remained unchanged. Combining the two procedural groups, the average annual mortality was 1.88 % (1998–2002) compared with 1.67 % (2003–2004)	Positive
Guru et al., 2006 [31]	To evaluate the differences in clinical outcomes observed during the transition from no reporting to confidential, and ultimately PR cards for CABG surgery in a public health system in Ontario	Cohort study for the same facility before and after the introduction of a PR mechanism	CABG surgery patients in Ontario Hospitals (1 September 1991–31 March 2002)	Ontario institution-level performance report cards on outcomes of CABG surgery	Cardiac mortality	The risk-adjusted 30-day mortality rate decreased 29 % from the era of no reporting (1991–1993) to confidential reporting (1994–1998). There was no further decrease with PR (1999–2001). In-hospital mortality fell significantly faster in Ontario during the period of confidential reporting than in other parts of Canada	Positive
Jha et al., 2006	To examine the impact of NYS CSRS fifteen years after its launch on cardiac surgery mortality	Cohort study for the same facility before and after the introduction of a PR mechanism	All New York Hospital cardiac surgeons performing CABG (1989–2002)	NYS CSRS	Cardiac mortality	Users who picked a top-performing hospital or surgeon from the latest available report had approximately half the chance of dying (risk-adjusted mortality rate = 1.59) as did those who picked a hospital or surgeon from the bottom quartile (risk-adjusted mortality rate = 2.78).	Positive
Hollenbeak et al., 2008 [33]	To assess effect of intensive PHC4 on hospital mortality for 6 high-frequency, high-mortality medical conditions	Cohort study among different facilities with and without PR mechanisms, over the same period of time	Cardiac surgery patients in Pennsylvania Hospitals (1997–2003)	PHC4	Cardiac, Respiratory, Neurologic and Sepsis mortality	Patients treated at hospitals subjected to intensive PR had significantly lower odds of in-hospital mortality when compared with similar patients treated at hospitals in environments with no PR or only limited reporting. The 2000–2003 in-hospital mortality OR for Pennsylvania patients versus non-Pennsylvania patients ranged from 0.59 to 0.79 across 6 clinical conditions. For the same comparison using the 1997–1999 period, OR ranged from 0.72 to 0.90.	Positive
Friedberg et al., 2009 [34]	To determine association of PR with over diagnosis of pneumonia, excessive antibiotic use, or inappropriate prioritization of patients with respiratory symptoms	Cohort study for the same facility before and after the introduction of a PR mechanism	Patients with respiratory symptoms in the National Hospital Ambulatory Medical Care Survey (2001–2005)	Hospital Quality Alliance data on antibiotic timing in pneumonia	Rates of pneumonia diagnosis, antibiotic use, and waiting times to see a physician	Public reporting of hospital antibiotic timing scores has not led to increased pneumonia diagnosis, antibiotic use, or change in patient prioritization. Comparing outcomes before and after antibiotic timing score reporting, there were no differences in rates of pneumonia diagnosis (10 % vs. 11 %) or antibiotic administration (34 % vs. 35 %).	None
Ryan, 2009 [35]	To evaluate the effects of the PHQID, a public quality reporting and P4P program, on Medicare patient mortality	Cohort study among different facilities with and without PR mechanisms, over the same period of time	Medicare patients with AMI, heart failure, pneumonia, or a CABG procedure from acute care hospitals (2000–2006).	PHQID program	Cardiac and Respiratory mortality	No evidence that the PHQID had a significant effect on risk-adjusted 30-day mortality for AMI, heart failure, pneumonia, or CABG.	None
Li et al., 2010 [36]	To evaluate the impact of PR by comparing CABG volume and mortality for hospitals and surgeons in the first year of state-mandated PR (2003), and with the most recent data available (2006)	Cohort study for the same facility before and after the introduction of a PR mechanism	Cardiac surgery patients from the California Hospital CABG Outcomes Reporting Program database for 2003 and 2006	California CABG Outcomes Reporting Program	Cardiac mortality	The statewide observed mortality declined from 2.90 % in 2003 to 2.22 % in 2006. Overall, the empiric odds ratio of operative death for 2006 patients was 24 % lower than for 2003 patients. Total CABG volume decreased from 2003 to 2006 by almost 27 %.	Positive
Werner et al., 2010 [37]	To estimate changes in cardiac and respiratory mortality, length of stay and readmission rate after Hospital Compare was initiated	Cohort study for the same facility before and after the introduction of a PR mechanism	Patients with AMI, heart failure, pneumonia from 3,476 acute care, nonfederal U.S. hospitals that publicly reported quality information on the CMS Hospital Compare Web site (2004–2006)	The Centers for Medicare and Medicaid Services and other health care organizations participate in the Hospital Quality Alliance	Cardiac and Respiratory mortality, length of stay, readmission rate	There was a decline in mortality rates (0.6 % points), lengths-of-stay (0.19 days), and readmission rates (0.5 % points) for acute myocardial infarction from 2004 to 2006. Changes in outcomes for heart failure and pneumonia were less consistent and smaller, when present at all.	Positive
Jha et al., 2012 [38]	To assess the long-term effect of the Medicare PHQID on cardiac and respiratory mortality at Premier versus Non Premier hospitals	Cohort study among different facilities with and without PR mechanisms, over the same period of time	Patients with AMI, CHF, CABG, pneumonia in New England Hospital (2003–2009)	Premier Healthcare Informatics program	Cardiac and Respiratory 30-day mortality	No evidence that the largest hospital-based pay-for-performance program led to a decrease in 30-day mortality. The rates of decline in mortality per quarter at Premier and Non Premier hospitals were also similar (0.04 and 0.04 %, respectively; and mortality remained similar after 6 years under the pay-for-performance system (11.82 % for Premier hospitals and 11.74 % for non-Premier hospitals)	None
Joynt et al., 2012 [39]	To evaluate PCI mortality in PR states versus non-reporting states in USA	Cohort study among different facilities with and without PR mechanisms, over the same period of time	Medicare patients admitted with acute MI to US acute care hospitals (2002–2010)	Mandatory state PR programs (NY, MA and PA) for PCI	Cardiac mortality	There were no differences in overall mortality among patients with acute MI in reporting vs non reporting states. In Massachusetts, odds of PCI for acute MI were comparable with odds in non reporting states prior to PR (40.6 % vs 41.8 %; OR, 1.00). Among Medicare beneficiaries with acute MI, the use of PCI was lower for patients treated in states with PR compared with patients treated in states without PR	Mixed
Renzi et al., 2012 [40]	To evaluate association between public reporting of hospital performance and PCI rates, hip fractures, cesarean deliveries in Lazio versus other regions of Italy	Cohort study for the same facility before and after the introduction of a PR mechanism and among different facilities with and without PR mechanisms over the same period of time	Patients with acute MI, hip fractures, and maternity patients discharged from any hospital within the Italian National Health Service (2006–2009)	Regional Outcome Evaluation Program P.Re. Val.E.	PCI rates, hip fractures, cesarean deliveries	In Lazio PCI within 48 h, changed from 22.49 to 29.43 % following reporting of the P.Re.Val.E results. In the other regions this proportion increased from 22.48 to 27.09 % during the same time period. Hip fractures operated on within 48 h increased from 11.73 to 15.78 % in Lazio, and not in other regions (from 29.36 to 28.57 %). Cesarean deliveries did not decrease in Lazio (from 34.57 to 35.30 %, and only slightly decreased in the other regions (from 30.49 to 28.11 %).	Positive
Ryan et al., 2012 [41]	To estimate the effect of Hospital Compare, Medicare’s PR initiative on 30-day mortality for heart attack, heart failure, and pneumonia	Cohort study for the same facility before and after the introduction of a PR mechanism	Medicare patients in USA Hospitals with heart attack, heart failure, pneumonia, stroke, gastrointestinal hemorrhage, and hip fracture (2000–2008)	Hospital Compare, Medicare’s PR initiative	Cardiac and Respiratory 30-day mortality	Hospitals that reported quality data under Hospital Compare had no reductions in mortality beyond existing trends for heart attack and pneumonia and led to a modest reduction in mortality for heart failure (RR = 0.92)	None
Linkin et al., 2013 [42]	To evaluate the association between state-legislated PR of hospital-acquired infection with infection control process	Cohort study among different facilities with and without PR mechanisms, over the same period of time	Patients from 137 eligible US hospitals in 35 states (2008–2011)	Medicare’s Hospital Compare website reports	Improvements in infection prevention	There is not estimated improvement in infection prevention program or hospital-acquired infection rates in hospitals in states legislating mandatory PR	None
McCabe et al., 2013 [43]	To evaluate the impact of PR of hospitals as negative outliers, on PCI risk adjusted mortality and case mix selection	Cohort study among different facilities with and without PR mechanisms, over the same period of time	Cardiac patients at all non-federally funded Massachusetts hospitals performing PCI (2003–2010)	National Cardiovascular Data Registry and Massachusetts Data Analysis Center model	In-hospital cardiac mortality	After public identification as a negative outlier institution, there was an 18 % relative reduction in predicted mortality among PCI patients at outlier institutions compared with non-outlier institutions	None
Marsteller et al., 2014 [44]	To evaluate mandatory reporting in participation and performance in reducing CLABSI in a national patient safety collaborative	Cohort study among different facilities with and without PR mechanisms, over the same period of time	Patients of intensive care units participating in the US national Comprehensive Unit-based Safety Program: Stop Bloodstream Infections (2009–2011)	Comprehensive Unit-based Safety Program: Stop Bloodstream Infections	CLABSI mortality	There was a reductions in CLABSI rates in the first 6 months compared with the units in states with no reporting requirement. During months 13–18, both state groups with mandatory PR of CLABSI showed a trend toward greater reduction in CLABSI compared with states with no requirement.	Positive
Wang et al., 2014 [45]	To evaluate the effect of publicly reporting performance data of medicine use on the injection prescribing rate	Cohort study for the same facility before and after the introduction of a PR mechanism	Effective electronic injection prescriptions in Primary healthcare institutions of Hubei province (China 2013–2014)	Database of electronic prescriptions of the local health bureau	Injection prescribing rates	PR led to a reduction of approximately 4 % in the injection prescribing rate four months after intervention (OR = 0.96). The intervention effect was inconsistent in each month after intervention, and it was most positive in the second month after intervention (OR = 0.90)	Positive
Yang et al., 2014 [46]	To evaluate the impact of PR on antibiotic prescribing for URTI in a sample of primary care institutions	Cohort study among different facilities with and without PR mechanisms, over the same period of time	URTI patients in Primary healthcare institutions of Hubei province (China 2013–2014)	Electronic health information system	Antibiotic prescribing	PR interventions reduced the incidence of oral antibiotic prescription (9 % point reduction adjusted RR = 39 %) and slowed down the increase of combined use of antibiotics for URTIs (7 % point reduction (adjusted RR = 36 %), while the use of injectable antibiotics remained unchanged. The intervention had little impact on the use of IV injections or infusions, or the total prescription expenditure	Mixed

*Abbreviations*: *AMI* Acute Myocardial Infarction, *CABG* Coronary Artery Bypass Graft, *CCSIP* California Cardiac Surgery and Intervention Project, *CHF* Congestive Heart Failure, *CHQC* Cleveland Health Quality Choice, *CLABSI* Central Line-Associated BloodStream Infections, *NYS CSRS* New York State Cardiac Surgery Reporting System, *P4P* Pay-for-Performance, *PCI* Percutaneous Coronary Intervention, *PHC4* Pennsylvania Health Care Cost Containment Council, *PHQID* Premier Hospital Quality Incentive Demonstration, *PR* Public Reporting, *URTI* Upper Respiratory Tract Infections

There were several clinical outcomes examined throughout the studies. Many investigated the effect of PR on patients’ mortality [14, 21–33, 35–39, 41, 43, 44]. Other aims in these studies included investigation of cardiac readmission to hospital [28, 37], antibiotic use, and waiting times to see a physician [34], injection prescribing rates [45], Percutaneous Coronary Intervention (PCI) rates, hip fractures operated on within 48 h, cesarean deliveries [40], change in CABG operations volume [14], improvements in infection prevention [42] and patient choices [36].

We included a total number of 27 studies in our systematic review that evaluated the effect of PR on clinical outcomes and the results are summarized in Table 1. Mainly, the effect of PR on clinical outcomes was positive. Fourteen studies reported positive results [14, 21–23, 25, 30–33, 36, 37, 40, 44, 45], nine reported not significant results [26, 27, 29, 34, 35, 38, 41–43], three studies reported mixed results [24, 39, 46], some positive and some negative or null, while one study reported a negative effect [28].

We used a GRADE derived approach to assess the quality of the included studies. The body of evidence in our review was characterized by a low quality level (see Additional file 1). Indeed, the study design was, in almost all of the studies, observational, and there were a number of limitations. When a pre-post approach at hospital level was used to assess the performance before and after the release of PR, there was no external control group for comparison. Moreover, there was no information on institutions and participants that were lost-to-follow-up during the study period. Also, the different outcomes, for institutions with and without PR, could be influenced by some characteristics not measured across the studies.

### Effects of public reporting on mortality

The effect of PR on mortality, as isolated clinical outcome, was evaluated throughout 22 studies [14, 21–33, 35–39, 41, 43, 44]. All studies were set in hospitals, in the US and Canada, and 19 of them were on cardiac patients and used PR data mostly from specific CSRS. A positive effect of PR on mortality was reported in 12 studies [14, 21–23, 25, 30–33, 36, 37, 44].

Two studies reported mixed effect of the PR on patients’ mortality. Baker et al. [24] demonstrated that, for most conditions, after the release of PR, risk-adjusted in-hospital mortality declined and mortality rate in the early post discharge period rose, while the 30-day mortality rate declined for heart failure and obstructive pulmonary disease and increased for stroke, while Joynt et al. [39] stated no differences in reporting versus non reporting states for overall mortality among patients with acute myocardial infarction (AMI), except among Medicare beneficiaries with AMI [39].

The only study in our review that reported a negative effect of PR was a cohort study from Dranove et al. [28], where the authors showed a statistically marginal evidence that after releasing the report cards, the average mortality rate in New York and Pennsylvania hospitals performing CABG increased by 0.45 percentage point on a base of 33 %. The remaining 7 studies reported non-significant effect of PR on mortality [26, 27, 29, 35, 38, 41, 43].

**Fig. 2:**
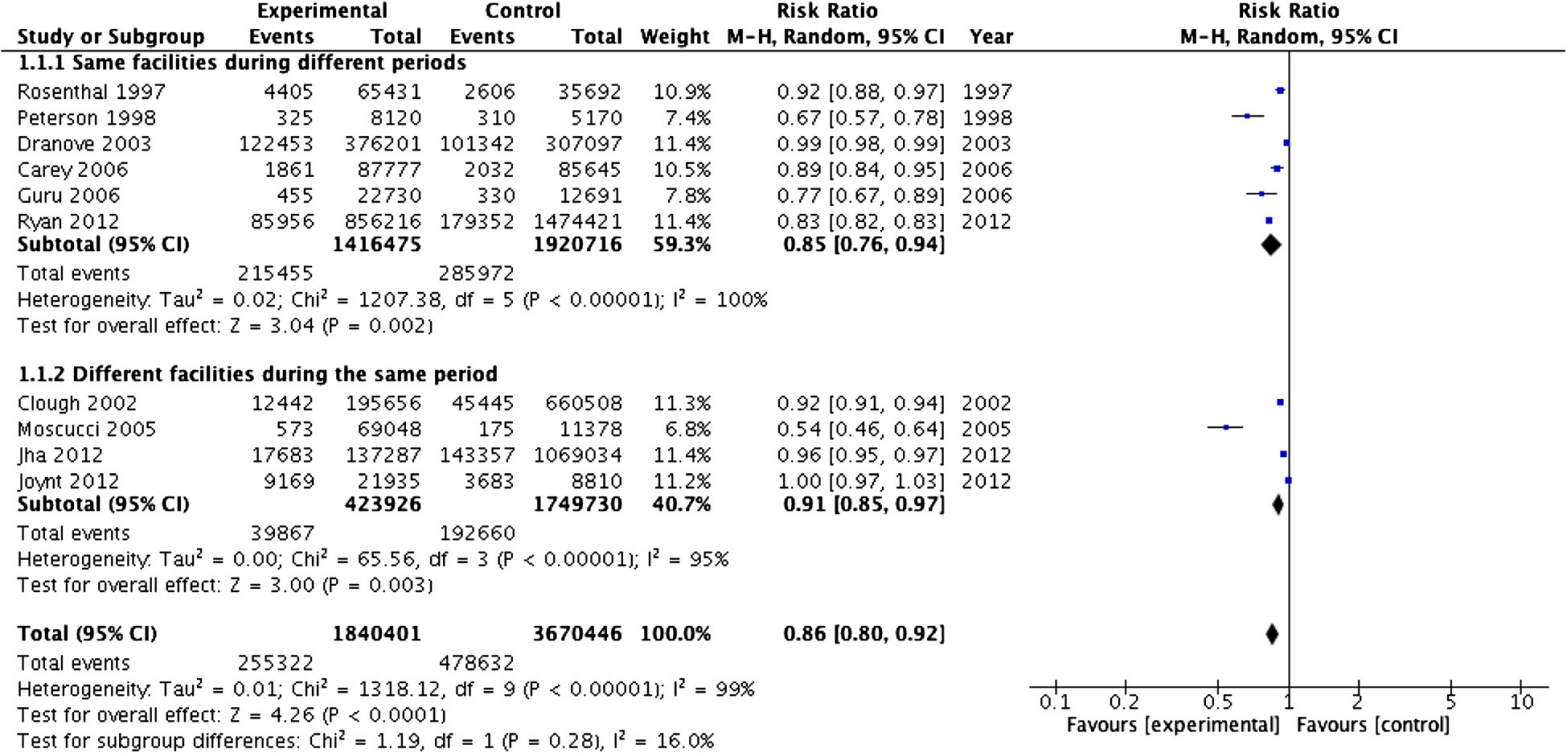
Meta-analysis of the PR effect on mortality as clinical outcome by facilities

Ten out of the 22 studies investigating mortality as an outcome [22, 23, 26, 28–31, 38, 39, 41] reported sufficient quantitative data to be pooled through meta-analysis. Overall, this analysis included a total of 1,840,401 experimental events and 3,670,446 control events. The meta-analysis resulted in a RR of 0.85 (95 % CI, 0.79-0.92) in a contest of high heterogeneity (*p* ≤ 0.0001; I^2^ = 99.1 %) (Fig. 2). Publication bias was not evident using the Egger’s test (*p* = 0.91). We also performed a one-way sensitivity analysis, where one by one study was omitted from the overall meta-analysis, but no significant change in risk estimates was noticed.

A subgroup analysis on mortality by study design was also carried out. The six publications [22, 23, 28, 30, 31, 41] reporting mortality rates in the same facilities during different periods showed a RR of 0.85 (95 % CI, 0.76-0.94) in a context of high heterogeneity (*p* < 0.0001; I^2^ = 100 %). Whilst, the four included studies [26, 29, 38, 39] recording mortality rates during the same period in different facilities showed a RR of 0.91 (95 % CI, 0.85-0.97) in a context of high heterogeneity I^2^ = 95 % (*p* < 0.0001) (Fig. 2). Test for subgroup differences resulted negative with a *p* value of 0.28.

**Fig. 3:**
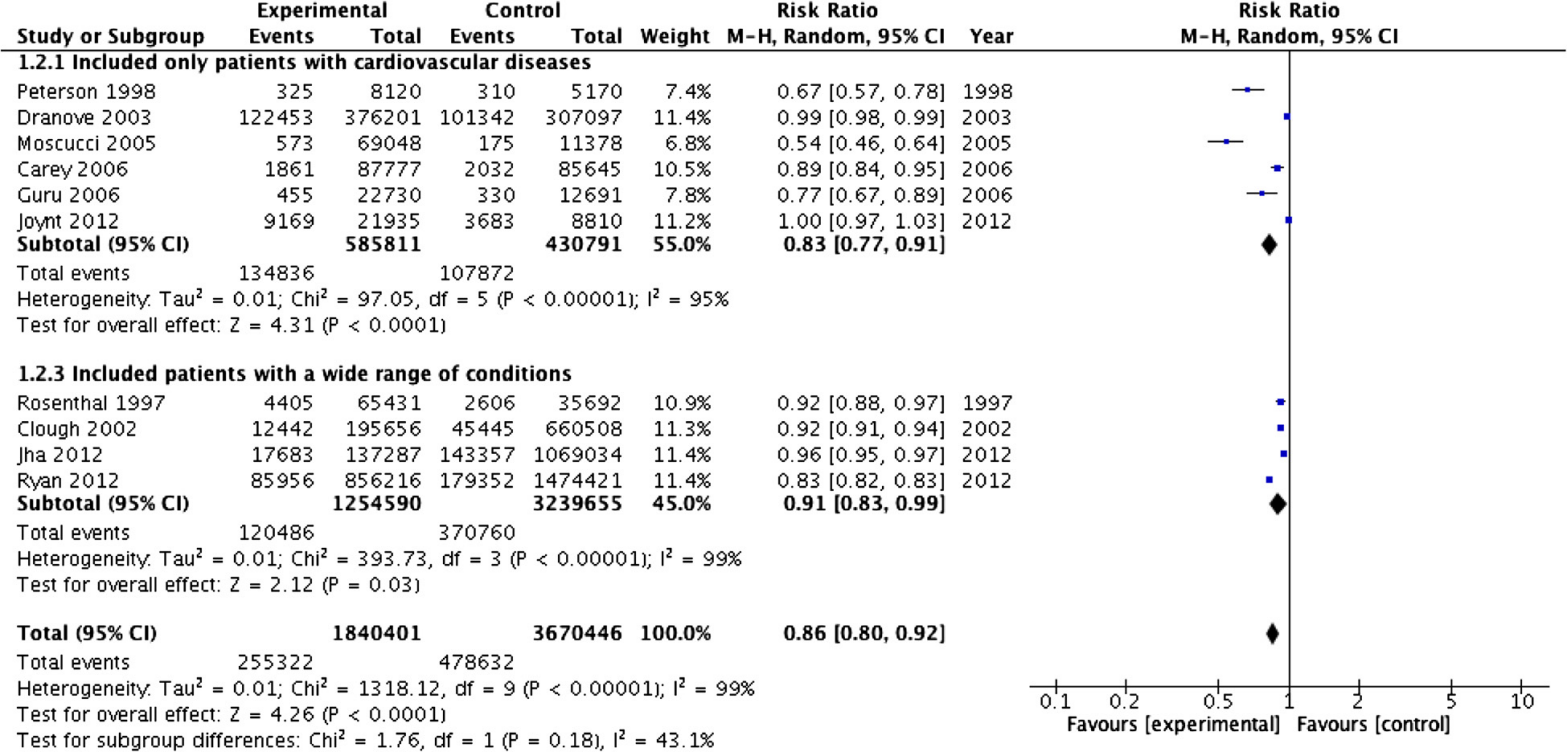
Meta-analysis of the PR effect on different mortality causes as clinical outcomes

Another subgroup analysis was performed by studies considering different mortality causes. Pooling the results from studies focused on mortality from cardiovascular disease, six studies were included [23, 28–31, 39] and a RR of 0.83 (95 % CI, 0.77-0.91) was calculated, with high heterogeneity (*p* < 0.0001; I^2^ = 95 %). For the subgroup of studies that included patients with a wide range of conditions [22, 26, 38, 41], a RR of 0.91 (95 % CI, 0.83-0.99) was obtained, with heterogeneity I^2^ = 99 % (*p* < 0.0001) (Fig. 3). Test for subgroup differences resulted negative with a *p* value of 0.18.

### Effects of public reporting on other clinical outcomes

Other clinical outcomes, beside mortality, were explored in 8 studies [28, 34, 37, 39, 40, 42, 45, 46]. In an Italian study performed by Renzi et al. [40], a pre-post evaluation of clinical outcomes in Lazio Region was done and also a comparative evaluation versus Italian Regions without comparable PR programs. The study demonstrated that in Lazio the PCI within 48 h from admission increased from 22.49 to 29.43 % following the reporting of the Regional Outcome Evaluation Program (P.Re. Val.E) results. In the other regions without comparable programs, during the same period, this proportion increased from 22.48 to 27.09 %. Hip fractures operated on within 48 h from admission increased in Lazio, while not in other regions with no PR. Cesarean deliveries did not decrease in Lazio (from 34.57 to 35.30 %), while only slightly decreased in the other regions (from 30.49 to 28.11 %) [40]. Joynt et al. [39] reported that after implementation of PR, odds of undergoing PCI in Massachusetts where PR was present decreased comparing with non-reporting states (41.1 % vs 45.6 %; OR = 0.81; 95 % CI = 0.47- 1.38; *P* = 0.03). Marsteller et al. [44] in one cohort study on patients from 1,046 adult intensive care units found reductions in central line-associated bloodstream infections (CLABSIs) rates in the first 6 months from the release of PR, compared to the units from the states with no reporting mechanisms. During months 13–18, groups with mandatory PR of data about CLABSI showed a trend toward greater reduction in CLABSI compared with states with no PR [44]. Hospital readmissions were evaluated in two studies. Dranove et al. [28] reported a negative effect of PR, with a significantly increased average rate of readmissions with heart failure by approximately 0.5 percentage point. On the other hand, more recently, Werner et al. [37] reported performance improvements for AMI in terms of declines in readmission rates beside lengths-of-stay and mortality rates.

Studies set up outside hospital settings [45, 46] addressed different clinical outcomes, such as antibiotic prescription and usage as well as injection prescribing rates, in primary healthcare institutions. Yang et al. [46] evaluated the impact of PR on antibiotic prescribing for upper respiratory tract infections (URTIs) demonstrating that PR interventions reduced the incidence of oral antibiotic prescription and slowed down the increase of combined use of antibiotics for URTIs in primary healthcare setting. According to Wang et al. [45], PR led to a reduction of approximately 4 % in the injection prescribing rate four months after intervention (OR = 0.96; 95 % CI: 0.94, 0.97), although with an inconsistent effect in each month after intervention.

## Discussion

The public release of hospital performance data has been recommended as one key strategy for stimulating improvement of quality of care by putting the focus on transparency and accountability of healthcare providers. Also, PR is expected to stimulate active patients/citizens participation by helping them make informed choices when choosing health care providers [47].

According to Berwick, PR can improve performance through 2 pathways. In the Improvement through Selection pathway, patients and providers could shift care from low-quality to high-quality hospitals by using the publicly disclosed reports of hospital performance quality and thereby stimulating quality improvement efforts for the benefits of market share. In the Improvement Through Changes in Care (or quality improvement) pathway, published performance data can identify areas in which providers had low accomplishment and help them to focus on improving performance, by appealing to their professionalism or their concern about reputation or direct market position. As well, it is more likely that quality improvement happens in combination of these two pathways [9].

Nowadays, PR is a more and more common health policy tool to stimulate and maintain quality improvement of health care. There is a growing international interest in providing the necessary information of clinical quality and performance of healthcare providers [48]. Many studies of PR performance data have been published so far, but available reviews of the association between clinical outcomes and public disclosure of performance data are limited.

In our systematic review on impact of PR on clinical outcomes, we identified 27 new articles published since 1994. These studies, which were mostly hospital-level and had medium global ratings, focused primarily on mortality rates and cardiac procedures. Ten studies found that mortality rates decreased after PR, while nine studies did not find a significant link between PR and improvement.

Most of the studies examined the impact of USA and Canada PR of mortality rates for cardiac surgery (CABG and PCI). Different reporting systems were evaluated across the publications. Many studies that we found, were focusing their research on the same group of reporting systems like NYS CSRS which is considered as a pioneer among PR systems, despite the fact that nowadays many PR systems are available on the market [14]. Also studies comparing effectiveness among different PR systems are lacking. Many studies used reporting systems based on cardiovascular outcomes, including surgical interventions, hospitalization and mortality. In our review, we found seven studies based on the NYS CSRS [14, 21, 23, 25, 28, 32, 39], four studies on Cleveland Health Quality Choice Program (CHQC) [22, 24, 26, 27], as well as Medicare’s PR initiative [35, 37, 41, 42]. Other studies used various state-level PR data sources. The majority of the studies (*N* = 25) were placed in hospital settings [14, 21–23, 26, 27, 29–38, 40–43], and two in primary healthcare institutions [45, 46].

As opposed to the studies performed in the hospital setting, little information is available from the literature regarding the effectiveness of PR in primary care settings. Translating knowledge and experience from one healthcare setting to others is a reasonable method for building the high-quality healthcare service everywhere [49]. Two studies included in this review [45, 46] demonstrated that PR can have an impact on medication prescription and usage in primary healthcare settings. The authors concluded that even though further clinical outcomes of such an impact were not investigated in their studies, a positive effect might be expected. To the best of our knowledge, our review is the first to tackle the effects of PR in primary care setting, so additional research is still needed to further investigate the mechanism by which public reporting takes effect in outside-hospital settings.

Our research, including 27 studies carried out in North America, Europe and China, was mainly conclusive with the previously published reviews of public reports, whereas the effect of the PR on clinical outcomes was in our qualitative and also, through meta-analysis technique, quantitative assessment positive for patients in the hospital setting and primary healthcare but with a low quality of the evaluated studies. In one of the first reviews about PR, Marshall et al. [50] showed improvement in health outcomes among 7 PR systems based in the USA. In 2008, a systematic review of 11 studies by Fung et al. [5], showed inconsistent association between PR and effectiveness on patient outcomes. They reported that studies of the effect of PR on outcomes provided mixed signals and that most of the evaluated studies were descriptive and had low global ratings thus limited strength of evidence. They also concluded that PR stimulated quality improvement activity in hospitals and yet did not affect hospital selection by patients and generally was limited to a few clinical areas, like cardiac surgery. In one recent report, Specchia et al. [51] performed a review on the use of publicly released performance data and concluded that the introduction of standard set of evidence based outcomes and performance measures at national level could reduce the pressure from the selection of performance measures to be disseminated through the PR and make health care providers aware of the importance of transparency and accountability. They also concluded that it would be very effective to link PR to Pay-for-Performance (P4P) systems by basing payments on outcome results and thereby supporting the quality improvement process.

### Strengths and limitations

This review has some strengths and limitations. The comprehensiveness, the focus on clinical outcomes and the attempt to provide a quantitative synthesis of the available evidence are strengths of this review. Our search, in fact, covered a wide time interval and a variety of settings including both hospitals and primary care institutions and results has been synthetized both qualitative and, for mortality outcome, quantitative through meta-analysis technique. However, because of the resulting heterogeneity, caution should be placed in interpreting the results of the quantitative synthesis made for mortality outcome.

Some studies [14, 21, 43] have also noted how, after the introduction of PR, structures starting from a lower quality level have a greater propensity to improve their quality compared to those starting from an already high level of quality. Difference in starting levels of quality between the different hospitals and institutions when introducing PR is also a source of heterogeneity among the studies included in our review.

Limitations are also present due to the observational nature of the included studies. Indeed, the studies which control and study cohorts were taken from the same facilities before and after the introduction of a PR mechanism may have overestimated the effect of PR, because of a technological improvement trend running parallel to the adoption of the PR [41].

Search strategy was also limited to English language studies published from 1991 and most of published evidence concern cardiovascular disease in hospital setting.

### Policy implications

PR can be considered as one of the key drivers for transparency and accountability in the public health field. Far from providing a quantitative estimate of the current use of PR, the experiences described in this paper can represent a framework of opportunities for changing the relationship between patients/customers and healthcare providers and as a tool to support policy makers in addressing and allocating resources according to the assessment of providers’ performances and the publication of their results which are accessible and understandable to all of the stakeholders, including patients/customers.

Several successful examples of stakeholder involvement in the processes deriving from PR have been described both in the United States [50] and in Europe, as in Netherlands [52] or Germany [53]. In particular WHO Regional Office for Europe suggested key considerations for a successful strategy to encourage providers in improving the quality of services, the accountability of processes, the identification of failures, and providing with the use of publicly reported quality information strengthening communication tools, supporting public health professionals and making clearer consequent decisions [54].

The decision-making is always a difficult process for patients. To obtain informed choices high levels of numeracy and literacy are needed and PR can be considered a useful instrument to face this issue. Therefore, policy makers should support good practices in Public Health especially those, such as PR, focused on increased patient awareness.

Moreover, health policies should be promoted with the aim to integrate different strategies pursuing quality and excellence in healthcare. For example, linking PR to P4P, and therefore to remuneration for incentives schemes based on performance data, would strengthen the competitiveness of the whole system, triggering at the same time a virtuous circle oriented to the increase of both appropriateness and continuous quality improvement of healthcare [51].

## Conclusions

The introduction of PR programs at different levels of the healthcare sector is a challenging but rewarding public health strategy. Existing research covering different clinical outcomes supports the idea that PR could, in fact, stimulate providers to improve healthcare quality.

Transparency and accountability resulting from PR implementation not only give patients those information tools customers commonly are able to access in many other sectors, but are key points in the process that make patients and citizens empowered protagonist of their care.

## Abbreviations

AHRQ, Agency for Healthcare Research and Quality; AMI, acute myocardial infarction; CABG, Coronary Artery Bypass Graft (CABG); CAHPS, Consumer Assessment of Healthcare Providers and Systems; CHQC, Cleveland Health Quality Choice Program; CLABSIs, central line-associated bloodstream infections; CRAG, Clinical Resource and Audit Group; HQID, Hospital Quality Incentive Demonstration program; NYS CSRS, New York State Cardiac Surgery Reporting System; P4P, pay-for-performance; PCI, Percutaneous Coronary Intervention; PR, Public Reporting; URTIs, upper respiratory tract infections (URTIs)

## Additional file

Additional file 1: Table reporting quality of the studies. (DOCX 58 kb)

## References

[1] Totten AM, Wagner J, Tiwari A, O’Haire C, Griffin J, Walker M. Closing the quality gap: revisiting the state of the science (vol. 5: public reporting as a quality improvement strategy). Evid Rep Technol Assess (Full Rep). 2012;5:1–645. PMC4781596 24422977

[2] Smith P, Mossialos E, Papanicolas I. Performance measurement for health system improvement: experiences, challenges and prospects. WHO Eur Minist Conf Heal Syst. 2008;1:1–22.

[3] Lansky D (2002). Improving quality through public disclosure of performance information. Health Aff.

[4] Hibbard JH (2012). What Can We Say about the impact of public reporting ? inconsistent. Ann Intern Med.

[5] Fung CH, Lim Y, Mattke S, Damberg C, Shekelle PG (2008). Systematic review: the evidence that publishing patient care performance data improves quality of care. Ann Intern Med.

[6] Werner RM (2005). The unintended consequences of publicly reporting quality information. JAMA.

[7] Colmers JM. Public reporting and transparency. Commonw Fund Comm a High Perform Heal Syst. 2007;1:1–16.

[8] Schneider E, Lieberman T (2001). Publicly disclosed information about the quality of health care: response of the US public. Qual Health Care.

[9] Berwick DM, James B, Coye MJ (2003). Connections between quality measurement and improvement. Med Care.

[10] Hibbard JH, Stockard J, Tusler M (2005). Hospital performance reports: impact on quality, market share, and reputation. Health Aff (Millwood).

[11] Marshall JJ, Zoghbi WA, Gillis AM (2013). Public reporting of cardiovascular care: an opportunity to shape the future. Catheter Cardiovasc Interv.

[12] Brown DL, Clarke S, Oakley J (2012). Cardiac surgeon report cards, referral for cardiac surgery, and the ethical responsibilities of cardiologists. J Am Coll Cardiol.

[13] Dehmer GJ, Drozda JP, Brindis RG, Masoudi FA, Rumsfeld JS, Slattery LE, Oetgen WJ (2014). Public reporting of clinical quality data: An update for cardiovascular specialists. J Am Coll Cardiol.

[14] Hannan EL, Kumar D, Racz M, Siu AL, Chassin MR (1994). New York State’s Cardiac Surgery Reporting System: four years later. Ann Thorac Surg.

[15] Mannion R, Goddard M (2001). Impact of published clinical outcomes data: case study in NHS hospital trusts. BMJ.

[16] Liberati A, Altman DG, Tetzlaff J, Mulrow C, Gøtzsche PC, Ioannidis JPA, Clarke M, Devereaux PJ, Kleijnen J, Moher D (2009). The PRISMA statement for reporting systematic reviews and meta-analyses of studies that evaluate healthcare interventions: explanation and elaboration. BMJ.

[17] Balshem H, Helfand M, Schünemann HJ, Oxman AD, Kunz R, Brozek J, Vist GE, Falck-Ytter Y, Meerpohl J, Norris S, Guyatt GH. GRADE guidelines: 3. Rating the quality of evidence. Journal of Clinical Epidemiology. 2011;64:401–406. 10.1016/j.jclinepi.2010.07.015 21208779

[18] DerSimonian R, Laird N (1986). Meta-analysis in clinical trials. Control Clin Trials.

[19] Higgins JPT, Thompson SG (2002). Quantifying heterogeneity in a meta-analysis. Stat Med.

[20] Egger M, Davey Smith G, Schneider M, Minder C (1997). Bias in meta-analysis detected by a simple, graphical test. BMJ.

[21] Dziuban SW, McIlduff JB, Miller SJ, Dal Col RH (1994). How a New York cardiac surgery program uses outcomes data. Ann Thorac Surg.

[22] Rosenthal GE, Quinn L, Harper DL (1997). Declines in hospital mortality associated with a regional initiative to measure hospital performance. Am J Med Qual.

[23] Peterson ED, DeLong ER, Jollis JG, Muhlbaier LH, Mark DB (1998). The effects of New York’s bypass surgery provider profiling on access to care and patient outcomes in the elderly. J Am Coll Cardiol.

[24] Baker DW, Einstadter D, Thomas CL, Husak SS, Gordon NH, Cebul RD (2002). Mortality trends during a program that publicly reported hospital performance. Med Care.

[25] Chassin MR (2002). Achieving and sustaining improved quality: lessons from New York State and cardiac surgery. Health Aff (Millwood).

[26] Clough JD, Engler D, Snow R, Canuto PE (2002). Lack of relationship between the Cleveland health quality choice project and decreased inpatient mortality in Cleveland. Am J Med Qual.

[27] Baker DW, Einstadter D, Thomas C, Husak S, Gordon NH, Cebul RD (2003). The effect of publicly reporting hospital performance on market share and risk-adjusted mortality at high-mortality hospitals. Med Care.

[28] Dranove D, Kessler D, McClellan M, Satterthwaite M (2003). Is more information better? the effects of “report cards” on health care providers. J Polit Econ.

[29] Moscucci M, Eagle KA, Share D, Smith D, De Franco AC, O’Donnell M, Kline-Rogers E, Jani SM, Brown DL (2005). Public reporting and case selection for percutaneous coronary interventions: an analysis from two large multicenter percutaneous coronary intervention databases. J Am Coll Cardiol.

[30] Carey JS, Danielsen B, Junod FL, Rossiter SJ, Stabile BE (2006). The California Cardiac Surgery and Intervention Project: evolution of a public reporting program. Am Surg.

[31] Guru V, Fremes SE, Naylor CD, Austin PC, Shrive FM, Ghali WA, Tu JV (2006). Public versus private institutional performance reporting: what is mandatory for quality improvement?. Am Heart J.

[32] Jha AK, Epstein AM (2006). The predictive accuracy of the New York State coronary artery bypass surgery report-card system. Health Aff (Millwood).

[33] Hollenbeak CS, Gorton CP, Tabak YP, Jones JL, Milstein A, Johannes RS (2008). Reductions in mortality associated with intensive public reporting of hospital outcomes. Am J Med Qual.

[34] Friedberg MW, Mehrotra A, Linder JA (2009). Reporting hospitals’ antibiotic timing in pneumonia: adverse consequences for patients?. Am J Manag Care.

[35] Ryan AM (2009). Effects of the premier hospital quality incentive demonstration on medicare patient mortality and cost. Health Serv Res.

[36] Li Z, Carlisle DM, Marcin JP, Castellanos LR, Romano PS, Young JN, Amsterdam EA (2010). Impact of public reporting on access to coronary artery bypass surgery: the California Outcomes Reporting Program. Ann Thorac Surg.

[37] Werner RM, Bradlow ET (2010). Public reporting on hospital process improvements is linked to better patient outcomes. Health Aff (Millwood).

[38] Jha AK, Joynt KE, Orav EJ, Epstein AM (2012). The long-term effect of premier pay for performance on patient outcomes. N Engl J Med.

[39] Joynt KE, Blumenthal DM, Orav EJ, Resnic FS, Jha AK (2012). Association of public reporting for percutaneous coronary intervention with utilization and outcomes among Medicare beneficiaries with acute myocardial infarction. JAMA.

[40] Renzi C, Sorge C, Fusco D, Agabiti N, Davoli M, Perucci CA (2012). Reporting of quality indicators and improvement in hospital performance: the P.Re.Val.E. Regional Outcome Evaluation Program. Health Serv Res.

[41] Ryan AM, Nallamothu BK, Dimick JB (2012). Medicare’s public reporting initiative on hospital quality had modest or no impact on mortality from three key conditions. Health Aff (Millwood).

[42] Linkin DR, Fishman NO, Shea JA, Yang W, Cary MS, Lautenbach E (2013). Public reporting of hospital-acquired infections is not associated with improved processes or outcomes. Infect Control Hosp Epidemiol.

[43] McCabe JM, Joynt KE, Welt FGP, Resnic FS (2013). Impact of public reporting and outlier status identification on percutaneous coronary intervention case selection in Massachusetts. JACC Cardiovasc Interv.

[44] Marsteller JA, Hsu Y-J, Weeks K (2014). Evaluating the impact of mandatory public reporting on participation and performance in a program to reduce central line-associated bloodstream infections: evidence from a national patient safety collaborative. Am J Infect Control.

[45] Wang X, Tang Y, Zhang X, Yin X, Du X, Zhang X (2014). Effect of publicly reporting performance data of medicine use on injection use: a quasi-experimental study. PLoS One.

[46] Yang L, Liu C, Wang L, Yin X, Zhang X (2014). Public reporting improves antibiotic prescribing for upper respiratory tract infections in primary care: a matched-pair cluster-randomized trial in China. Health Res Policy Syst.

[47] Faber M, Bosch M, Wollersheim H, Leatherman S, Grol R (2009). Public reporting in health care: how do consumers use quality-of-care information? A systematic review. Med Care.

[48] Mannion R, Goddard M: Public disclosure of comparative clinical performance data: Lessons from the Scottish experience. Journal of Evaluation in Clinical Practice. 2003;9:277–286. 10.1046/j.1365-2753.2003.00388.x 12787191

[49] Ledikwe JH, Grignon J, Lebelonyane R, Ludick S, Matshediso E, Sento BW, Sharma A, Semo BW (2014). Improving the quality of health information: a qualitative assessment of data management and reporting systems in Botswana. Health Res Policy Syst.

[50] Marshall MN, Shekelle PG, Leatherman S, Brook RH (2000). The public release of performance data: what do we expect to gain? A review of the evidence. JAMA.

[51] Specchia ML, Veneziano MA, Cadeddu C, Ferriero AM, Capizzi S, Ricciardi W (2012). Peer pressure and public reporting within healthcare setting: improving accountability and health care quality in hospitals. Ig Sanita Pubbl.

[52] Delnoij DM, Rademakers JJ, Groenewegen PP (2010). The Dutch consumer quality index: an example of stakeholder involvement in indicator development. BMC Health Serv Res.

[53] Büscher A (2010). Public reporting, expert standards and indicators. Different routes to improve the quality of German long-term care. Eurohealth (Lond).

[54] Rodrigues R, Trigg L, Schmidt AE, Leichsenring K. The public gets what the public wants: Experiences of public reporting in long-term care in Europe. Health Policy. 2014;116:84-94. 10.1016/j.healthpol.2013.12.012 24461213

