# Supplementary material for: The impact of Public Reporting on clinical outcomes: a systematic review and meta-analysis
Source: BMC Health Serv Res. 2016 Jul 22;16:296. doi: 10.1186/s12913-016-1543-y (PMC4957420; doi:10.1186/s12913-016-1543-y)
Supplement: Additional file 1: — Table reporting quality of the studies. (DOCX 58 kb) [file 12913_2016_1543_MOESM1_ESM.docx]

| **First author, year of the study** |  | **Quality of the studies (GRADE Scale)** | | | | | | | | |
| --- | --- | --- | --- | --- | --- | --- | --- | --- | --- | --- |
|  |  | **Design** | **Risk of Bias** | **Consistency** | **Directness** | **Precision** | **Publication bias** | **Effect** | **Dose-Response** | **Residual confounding** |
| Hannan et al, 1994 (1) | **Characteristic** | Observational study | Minimal | Consistent | Direct | Precise | Not likely | Small (some statistically significant) | Minimal | Potential to increase or decrease effect |
|  | **Effect on quality** | Low | No change | No change | No change | No change | No change | No change | No change | No change |
| Dziuban et al, 1994 (2) | **Characteristic** | Observational study | Minimal | Consistent | Indirect | Precise | Not likely | Small | Not shown | Potential to increase or decrease effect |
|  | **Effect on quality** | Low | No change | No change | No change | No change | No change | No change | No change | No change |
| Rosenthal et al, 1997 (3) | **Characteristic** | Observational study | Minimal | Consistent | Indirect | Precise | Not likely | Small (some statistically significant) | Not shown | Potential to increase or decrease effect |
|  | **Effect on quality** | Low | No change | No change | No change | No change | No change | No change | No change | No change |
| Peterson et al, 1998 (4) | **Characteristic** | Observational study | Minimal | Consistent | Direct | Precise | Not likely | Small (some statistically significant) | Small | Potential to increase or decrease effect |
|  | **Effect on quality** | Low | No change | No change | No change | No change | No change | Increase | No change | No change |
| Chassin, 2002 (5) | **Characteristic** | Observational study | Minimal | Consistent | Direct | Precise | Not likely | Moderate | Small | Potential to increase or decrease effect |
|  | **Effect on quality** | Low | No change | No change | No change | No change | No change | Increase | No change | No change |
| Clough et al, 2002 (6) | **Characteristic** | Observational study | Minimal | Consistent | Direct | Precise | Not likely | Small | Very minimal | Potential to increase or decrease effect |
|  | **Effect on quality** | Low | No change | No change | No change | No change | No change | No change | No change | No change |
| Baker et al, 2002 (7) | **Characteristic** | Observational study | Minimal | Consistent | Direct | Precise | Not likely | Small (few statistically significant) | None | Potential to increase or decrease effect |
|  | **Effect on quality** | Low | No change | No change | No change | No change | No change | No change | No change | No change |
| Baker et al, 2003 (8) | **Characteristic** | Observational study | Minimal | Consistent | Direct | Precise | Not likely | Small (few statistically significant) | Small | Potential to increase or decrease effect |
|  | **Effect on quality** | Low | No change | No change | No change | No change | No change | No change | No change | No change |
| Dranove et al, 2003 (9) | **Characteristic** | Observational study | Minimal | Consistent (inconsistencies explained) | Direct | Precise | Not likely | Small | Moderate | Some potential |
|  | **Effect on quality** | Low | No change | No change | No change | No change | No change | No change | Increase | No change |
| Moscucci et al, 2005 (10) | **Characteristic** | Observational study | Multiple sources identified | Consistent | Direct | Precise | Not likely | Some statistically significant | Not shown | Potential to increase or decrease |
|  | **Effect on quality** | Low | Decrease | No change | No change | No change | No change | Increase | No change | No change |
| Carey et al., 2006 (11) | **Characteristic** | Observational study | Minimal | consistent | Direct | Precise | Not likely | Small | Association suggested | None |
|  | **Effect on quality** | Low | No change | No change | No change | No change | No change | No change | Increase | No change |
| Guru et al, 2006 (12) | **Characteristic** | Observational study | Minimal | consistent | Direct | Precise | Not likely | Small | Nearly none | Minimal |
|  | **Effect on quality** | Low | No change | No change | No change | No change | No change | No change | No change | No change |
| Jha et al, 2006 (13) | **Characteristic** | Observational study | Minimal | Consistent | Direct | Slightly less precise (small numbers) | Not likely | Small | Not significant | Potential to increase or decrease |
|  | **Effect on quality** | Low | No change | No change | No change | Decrease | No change | No change | Increase | No change |
| Hollenbeak et al, 2008 (14) | **Characteristic** | Observational study | Minimal | Consistent | Direct | Precise | Not likely | Small | Small | Potential to increase or decrease |
|  | **Effect on quality** | Low | No change | No change | No change | No change | No change | No change | Increase | No change |
| Ryan, 2009 (15) | **Characteristic** | Observational study | Small potential | Consistent | Direct | Slightly less precise (limited amount of patient/hospital data) | Not likely | None | None | Potential to increase or decrease effect |
|  | **Effect on quality** | Low | No change | No change | No change | Decrease | No change | No change | No change | No change |
| Friedberg et al, 2009 (16) | **Characteristic** | Observational study | Minimal | Consistent | Direct | Precise | Not likely | very minimal | Nearly none | Potential to increase or decrease effect |
|  | **Effect on quality** | Low | No change | No change | No change | No change | No change | No change | No change | No change |
| Werner et al, 2010 (17) | **Characteristic** | Observational study | Minimal | Consistent | Direct | Precise | Not likely | Small (Modest) | Not significant | Potential to increase or decrease effect |
|  | **Effect on quality** | Low | No change | No change | No change | No change | No change | No change | No change | No change |
| Li et al, 2010 (18) | **Characteristic** | Observational study | Minimal | Consistent | Direct | Precise | Not likely | Moderate | Some statistically significant | Potential to increase or decrease effect |
|  | **Effect on quality** | Low | No change | No change | No change | No change | No change | Increase | No change | No change |
| Jha et al, 2012 (19) | **Characteristic** | Observational study | Minimal | Consistent | Direct | Precise | Not likely | None | None found | Potential to increase or decrease effect |
|  | **Effect on quality** | Low | No change | No change | No change | No change | No change | No change | No change | No change |
| Joynt et al, 2012 (20) | **Characteristic** | Observational study | Minimal | Consistent | Direct | Precise | Not likely | Moderate | Significant association indicated | Potential to increase or decrease effect |
|  | **Effect on quality** | Low | No change | No change | No change | No change | No change | Increase | No change | No change |
| Renzi et al, 2012 (21) | **Characteristic** | Observational study | Minimal | Consistent | Direct | Precise | Not likely | Some statistically significant | Association suggested | Potential to increase or decrease effect |
|  | **Effect on quality** | Low | No change | No change | No change | No change | No change | Increase | No change | No change |
| Ryan et al, 2012 (22) | **Characteristic** | Observational study | Minimal | Consistent | Direct | Precise | Not likely | very small | None | Potential to increase or decrease effect |
|  | **Effect on quality** | Low | No change | No change | No change | No change | No change | No change | No change | No change |
| Linkin et al, 2013 (23) | **Characteristic** | Observational study | Minimal | Consistent | Direct | Precise | Not likely | None | Not shown | Potential to increase or decrease effect |
|  | **Effect on quality** | Low | No change | No change | No change | No change | No change | No change | No change | No change |
| McCabe et al, 2013 (24) | **Characteristic** | Observational study | Minimal | Consistent | Direct | Precise | Not likely | Small (some statistically significant) | very small | Potential to increase or decrease effect |
|  | **Effect on quality** | Low | No change | No change | No change | No change | No change | No change | No change | No change |
| Marsteller et al, 2014 (25) | **Characteristic** | Observational study | Minimal | Consistent | Direct | Precise | Not likely | small | small | Potential to increase or decrease effect |
|  | **Effect on quality** | Low | No change | No change | No change | No change | No change | No change | No change | No change |
| Yang et al, 2014 (26) | **Characteristic** | Experimental study | Minimal | Consistent | Direct | Precise | Not likely | Small (few statistically significant) | Small | Potential to increase or decrease effect |
|  | **Effect on quality** | High | No change | No change | No change | No change | No change | Increase | No change | No change |
| Wang et al, 2014 (27) | **Characteristic** | Observational study | Minimal | Consistent (possible inconsistencies accounted for) | Direct | Precise | Not likely | Moderate | Some statistically significant | Potential to increase or decrease effect |
|  | **Effect on quality** | Low | No change | No change | No change | No change | No change | Increase | No change | No change |

References

1. Hannan EL, Kumar D, Racz M, Siu AL, Chassin MR. New York state’s cardiac surgery reporting system: Four years later. Ann Thorac Surg. The Society of Thoracic Surgeons; 1994;58(6):1852–7.

2. Dziuban SW, McIlduff JB, Miller SJ, Dal Col RH. How a New York cardiac surgery program uses outcomes data. Ann Thorac Surg. The Society of Thoracic Surgeons; 1994 Dec;58(6):1871–6.

3. Rosenthal GE, Quinn L, Harper DL. Declines in hospital mortality associated with a regional initiative to measure hospital performance. Am J Med Qual. 1997 Jan;12(2):103–12.

4. Peterson ED, DeLong ER, Jollis JG, Muhlbaier LH, Mark DB. The effects of New York’s bypass surgery provider profiling on access to care and patient outcomes in the elderly. J Am Coll Cardiol. 1998 Oct;32(4):993–9.

5. Chassin MR. Achieving and sustaining improved quality: Lessons from New York state and cardiac surgery. Health Aff. 2002;21(4):40–51.

6. Clough JD, Engler D, Snow R, Canuto PE. Lack of relationship between the Cleveland Health Quality Choice project and decreased inpatient mortality in Cleveland. Am J Med Qual. 2002;17(2):47–55.

7. Baker DW, Einstadter D, Thomas CL, Husak SS, Gordon NH, Cebul RD. Mortality trends during a program that publicly reported hospital performance. Med Care [Internet]. 2002;40(10):879–90. Available from: http://www.ncbi.nlm.nih.gov/pubmed/12395022

8. Baker DW, Einstadter D, Thomas C, Husak S, Gordon NH, Cebul RD. The effect of publicly reporting hospital performance on market share and risk-adjusted mortality at high-mortality hospitals. Med Care. 2003 Jun;41(6):729–40.

9. Dranove D, Kessler D, McClellan M, Satterthwaite M. Is More Information Better? The Effects of “Report Cards” on Health Care Providers. J Polit Econ. 2003;111(3):555–88.

10. Moscucci M, Eagle K a, Share D, Smith D, De Franco AC, O’Donnell M, et al. Public reporting and case selection for percutaneous coronary interventions: an analysis from two large multicenter percutaneous coronary intervention databases. J Am Coll Cardiol. 2005 Jun;45(11):1759–65.

11. Carey JS, Danielsen B, Junod FL, Rossiter SJ, Stabile BE. The California cardiac surgery and intervention project: Evolution of a public reporting program. Am Surg. 2006;72(10):978–83.

12. Guru V, Fremes SE, Naylor CD, Austin PC, Shrive FM, Ghali WA, et al. Public versus private institutional performance reporting: What is mandatory for quality improvement? Am Heart J. 2006;152(3):573–8.

13. Jha AK, Epstein AM. The predictive accuracy of the New York State coronary artery bypass surgery report-card system. Health Aff. 2006;25(3):844–55.

14. Hollenbeak CS, Gorton CP, Tabak YP, Jones JL, Milstein A, Richard S, et al. Reductions in Mortality Associated Hospital Outcomes. 2008;

15. Ryan AM. Effects of the Premier Hospital Quality Incentive Demonstration on Medicare patient mortality and cost. Health Serv Res. 2009 Jun;44(3):821–42.

16. Friedberg MW, Mehrotra A, Linder JA. Reporting hospitals’ antibiotic timing in pneumonia: adverse consequences for patients? Am J Manag Care. 2009;15(2):137–44.

17. Werner RM, Bradlow ET. Public reporting on hospital process improvements is linked to better patient outcomes. Health Aff. 2010;29(7):1319–24.

18. Li Z, Carlisle DM, Marcin JP, Castellanos LR, Romano PS, Young JN, et al. Impact of public reporting on access to coronary artery bypass surgery: the California Outcomes Reporting Program. Ann Thorac Surg. Elsevier Inc.; 2010 Apr;89(4):1131–8.

19. Jha AK, Joynt KE, Orav EJ, Epstein AM. The long-term effect of premier pay for performance on patient outcomes. N Engl J Med. 2012 Apr;366(17):1606–15.

20. Joynt KE, Blumenthal DM, Orav EJ, Resnic FS, Jha AK. Association of public reporting for percutaneous coronary intervention with utilization and outcomes among Medicare beneficiaries with acute myocardial infarction. JAMA. 2012;308(14):1460–8.

21. Renzi C, Sorge C, Fusco D, Agabiti N, Davoli M, Perucci C a. Reporting of quality indicators and improvement in hospital performance: the P.Re.Val.E. Regional Outcome Evaluation Program. Health Serv Res. 2012 Oct;47(5):1880–901.

22. Ryan AM, Nallamothu BK, Dimick JB. Medicare’s public reporting initiative on hospital quality had modest or no impact on mortality from three key conditions. Health Aff (Millwood). 2012 Mar;31(3):585–92.

23. Linkin DR, Fishman NO, Shea JA, Yang W, Cary MS, Lautenbach E. Public reporting of hospital-acquired infections is not associated with improved processes or outcomes. Infect Control Hosp Epidemiol [Internet]. 2013;34(8):844–6. Available from: http://www.pubmedcentral.nih.gov/articlerender.fcgi?artid=3979462&tool=pmcentrez&rendertype=abstract

24. McCabe JM, Joynt KE, Welt FGP, Resnic FS. Impact of public reporting and outlier status identification on percutaneous coronary intervention case selection in Massachusetts. JACC Cardiovasc Interv. 2013 Jun;6(6):625–30.

25. Marsteller JA, Hsu Y-J, Weeks K. Evaluating the impact of mandatory public reporting on participation and performance in a program to reduce central line-associated bloodstream infections: evidence from a national patient safety collaborative. Am J Infect Control. 2014 Oct;42(10 Suppl):S209–15.

26. Yang L, Liu C, Wang L, Yin X, Zhang X. Public reporting improves antibiotic prescribing for upper respiratory tract infections in primary care: a matched-pair cluster-randomized trial in China. Health Res Policy Syst. 2014 Jan;12(1):61.

27. Wang X, Tang Y, Zhang X, Yin X, Du X, Zhang X. Effect of publicly reporting performance data of medicine use on injection use: a quasi-experimental study. PLoS One. 2014 Jan;9(10):e109594.
